# A new ALK isoform transported by extracellular vesicles confers drug resistance to melanoma cells

**DOI:** 10.1186/s12943-018-0886-x

**Published:** 2018-10-05

**Authors:** Giulia Cesi, Demetra Philippidou, Ines Kozar, Yeoun Jin Kim, Francois Bernardin, Guillaume Van Niel, Anke Wienecke-Baldacchino, Paul Felten, Elisabeth Letellier, Sonja Dengler, Dorothee Nashan, Claude Haan, Stephanie Kreis

**Affiliations:** 1 Life Sciences Research Unit, University of Luxembourg, Belvaux, Luxembourg; 2 NantOmics, Rockville, USA; 3 Luxembourg Institute of Health, Luxembourg, Luxembourg; 4 Institute Curie, PSL Research University, CNRS UMR144, Paris, France; 5 Center for Psychiatry and Neuroscience, Hopital Saint-Anne, Université Descartes, INSERM U894, Paris, France; 6 Klinikum Dortmund, Dortmund, Germany

**Keywords:** Melanoma, ALK, Extracellular vesicles, Drug resistance, Kinase inhibitors

## Abstract

**Background:**

Drug resistance remains an unsolved clinical issue in oncology. Despite promising initial responses obtained with BRAF and MEK kinase inhibitors, resistance to treatment develops within months in virtually all melanoma patients.

**Methods:**

Microarray analyses were performed in BRAF inhibitor-sensitive and resistant cell lines to identify changes in the transcriptome that might play a role in resistance. siRNA approaches and kinase inhibitors were used to assess the involvement of the identified Anaplastic Lymphoma Kinase (ALK) in drug resistance. The capability of extracellular vesicles (EVs) to transfer drug resistant properties was investigated in co-culture assays.

**Results:**

Here, we report a new mechanism of acquired drug resistance involving the activation of a novel truncated form of ALK. Knock down or inhibition of ALK re-sensitised resistant cells to BRAF inhibition and induced apoptosis. Interestingly, truncated ALK was also secreted into EVs and we show that EVs were the vehicle for transferring drug resistance.

**Conclusions:**

To our knowledge, this is the first report demonstrating the functional involvement of EVs in melanoma drug resistance by transporting a truncated but functional form of ALK, able to activate the MAPK signalling pathway in target cells. Combined inhibition of ALK and BRAF dramatically reduced tumour growth in vivo. These findings make ALK a promising clinical target in melanoma patients.

**Electronic supplementary material:**

The online version of this article (10.1186/s12943-018-0886-x) contains supplementary material, which is available to authorized users.

## Background

Melanoma is generally associated with poor outcome once metastatic disease stages have been reached. Compared to other solid cancers, this most aggressive form of skin cancer exhibits an extremely high prevalence of somatic mutations [1, 2], which is almost entirely attributable to UV light exposure. Despite this high genetic heterogeneity, 40–60% of melanoma patients carry mutations in the Ser/Thr-kinase BRAF (most often V600E), which renders the BRAF kinase and the downstream MAPK signalling pathway constitutively active [3]. The introduction of specific kinase inhibitors for melanoma patients carrying this BRAF mutation has revolutionised melanoma care. In 2011, BRAF inhibitors were FDA-approved showing convincing results at first [4, 5] and since 2015 a combined inhibition of BRAF and MEK kinases is recommended [6, 7], which has increased median survival from 18.7 to 25.1 months [8, 9]. However, despite these unprecedented clinical responses, drug resistance arises rapidly within 3–12 months [10, 11] leaving as only treatment options chemotherapy and in some cases immunotherapy. Most often, acquired resistance is driven by secondary mutations, which re-activate the MAPK signalling pathway resuming rapid proliferation.

Anaplastic lymphoma kinase (ALK) is a receptor tyrosine kinase that is normally involved in the development of the nervous system [12]. In differentiated tissues, ALK can be activated by translocations or mutations making it an oncogene in a variety of malignancies, such as non-small cell lung cancer, anaplastic large cell lymphoma, neuroblastoma and many more [13]. Additionally, in 2015, Wiesner and colleagues identified in 11% of melanoma tissues a truncated ALK transcript starting from intron 19 and resulting in a smaller protein, which was shown to be oncogenic [14].

Here, we identified the overexpression of a novel truncated form of ALK, named ALK^RES^ in the hereafter, as new mechanism driving acquired drug resistance in melanoma cells. In particular, we demonstrate that treatment of the ALK^RES^-expressing resistant melanoma cells with siRNA or ALK inhibitors in combination with either BRAF or MEK inhibitors, leads to efficient cell growth suppression and apoptosis, suggesting this combination to be an interesting clinical option for patients harbouring both BRAF^V600E^ and expressing ALK^RES^, especially as more specific ALK inhibitors become available. Moreover, we show for the first time that the overexpressed ALK^RES^ is secreted into extracellular vesicles (EVs) and is transferred to sensitive, ALK-negative melanoma cells. There, ALK^RES^ is functional in activating the MAPK signalling pathway and thus is involved in transferring of drug resistance. Finally, the combination of BRAF and ALK inhibitor treatments of mice bearing ALK-positive melanoma tumours dramatically reduced tumour volumes, making ALK an exciting clinical target in melanoma patients.

## Methods

### Inhibitors

All inhibitors used in this study were purchased from Selleckchem and were dissolved in DMSO at a concentration of 10 mM and stored at − 20 °C.

### Cell lines and cell culture

A375 melanoma cells were purchased from ATCC and cultured as previously described [15]. Drug-resistant clones were generated by culturing parental A375 cells in presence of 1 μM PLX4032 for 6–8 weeks. 20 different clones were picked and grown independently under constant PLX4032 treatment. The clone A375X1 was selected for further experiments.

### Microarray analysis

Total RNA was extracted with the miRNeasy mini kit (Qiagen) in triplicates following the manufacturer’s instructions. RNA quality was further assessed using the Agilent 2100 Bioanalyzer (Agilent Technologies). Microarray analyses were performed at the Luxembourg Institute of Health (LIH) by using the Affymetrix HuGene 2.0 ST platform as described previously [15]. The raw microarray data are accessible in the ArrayExpress database (https://www.ebi.ac.uk/arrayexpress/) under the accession number E-MTAB-6596.

### 5’RACE, sequencing of amplified products and PCR

5’RACE was performed according to the manufacturer’s instructions using the GeneRacer™ kit (Invitrogen) and ALK specific primers binding to exon 21 and to the junction between exon 24 and 25 were designed. The final product was sequenced at GATC Biotech (Konstanz, Germany). In addition, ALK was fully sequenced.

PCR amplification of both ALK and the fusion between MMLV and ALK were performed using specific primers. All primer sequences are listed in Additional file 1: Table S1.

### Quantitative PCR

Total RNA was extracted using the Quick-RNA™ miniprep kit (Zymo Research) according to the manufacturer’s instructions and the concentration and quality was determined using a NanoDrop Spectrophotometer. Quantitative real time qPCR was performed as described previously [15]. ALK primers listed in Additional file 1: Table S1.

### ALK immunoprecipitation

ALK was precipitated from lysates of A375X1 cells. Cells were lysed in RIPA buffer and incubated with ALK antibody (1:100) overnight at 4 °C on an overhead shaker. The next day, lysates were incubated with protein G sepharose™ (GE Healthcare), which was previously washed with the lysis buffer, for 1 h at 4 °C on an overhead shaker. After three washing steps, the protein was released by heat treatment in 2× Laemmli buffer and separated by SDS-PAGE.

### Small interfering RNAs and transfection

Three different ALK siRNAs were obtained from GE Dharmacon (ON-TARGETplus Human) (Additional file 1: Table S2). siRNA transfections were performed using 1.5 μl Lipofectamine RNAiMAX (Invitrogen) per reaction according to the manufacturer’s instructions. The final concentration of both ALK siRNA and scrambled control was 100 nM. siRNA transfections were performed 24 h prior to 48 or 72 h incubation with PLX4032 (1 μM), Trametinib (5 nM) or MK2206 (1 μM).

### Western blot analyses and antibodies

Cell lysis and Western blot analysis were performed as described previously [16, 17]. The following antibodies were used: phospho-ERK1/2, phospho-AKT, phospho-ALK and ALK (from Cell signaling), ERK1/2, tot-AKT and α-tubulin (from Santa Cruz), CD9 and CD81 (from System Biosciences) and TSG101 (from Abcam).

### Real-time proliferation assays

25 X 10^3^ cells/well of A375X1 melanoma cells were seeded in 24-well plates and 24 h later treated with both scrambled and ALK siRNA. Next, cells were incubated with PLX4032 (1 μM), Trametinib (5 nM) and MK2206 (1 μM). Cellular growth was monitored in the IncuCyte ZOOM live cell microscope (Essen BioScience) and images were taken in phase contrast every 3 h for a total of 90 h.

### Dose-response analysis of kinase inhibitors

Black 96-well μclear plates (Greiner) were used. In case of ALK inhibitors, 5000 cells/well of resistant A375X1 cells were seeded in RPMI medium. In order to determine the dose-response, kinase inhibitors were serially diluted at a ratio of 1:3, starting at 10 μM for Crizotinib and ASP3026 and starting at 1 μM for Ceritinib, in a total reaction volume of 100 μl. A blank control (RPMI medium only), as well as an untreated control were included for each cell line. For dose-response to vemurafenib, 3500 cells/well of resistant A375X1 cells were seeded and pre-treated with 1 μM of Crizotinib and ASP3026 and 100 nM of Ceritinib. 24 h after the pre-treatment, vemurafenib was serially diluted at a ratio of 1:3, starting at 10 μM and added to the cells. For drug resistance transfer, 1000 cells/well of sensitive A375 were seeded in 100 μl of RPMI medium. The day after, EVs at a concentration of 10 μg/ml were added to the cells. 24 h later, does-response to vemurafenib was performed.

For all experiments, cell viability was measured 72 h later using the CyQuant proliferation assay. Fluorescence intensity was measured using the microplate reader CLARIOstar^R^ (BMG-LABTECH). The blank corrected values were exported as Microsoft Excel files and analysed. Experiments were performed in technical and biological triplicates. Dose-response curves were generated using GraphPad Prism 5.

### Caspase-3 activity assay

To measure apoptosis in A375 and A375X1 cells, 20000 cells/well were seeded in black 96-well μclear plates and treated with 1 μM or 100 nM of single or combined inhibitors (PLX4032 or ALK inhibitors). Cells treated with etoposide (200 μM) were included as an internal positive control for apoptosis. 24 h later, cells were lysed with a lysis buffer containing dithiothreitol (6 mM) and DEVD-AFC substrate (AFC: 7-amino-4-trifluoromethyl coumarin) (Alfa Aesar) for 30 min at 37 °C. Upon cleavage of the substrate by caspases, free AFC emits fluorescence, which can be quantified using a microplate reader (400 nm excitation and 505 nm emission). Additionally, we included a blank control (RPMI medium only), an untreated control as well as a negative control represented by cells treated with DEVD-CHO (Alfa Aesar), a synthetic tetrapeptide inhibitor for Caspase-3. Fluorescence intensity was measured using the microplate reader CLARIOstar^R^ (BMG-LABTECH). The DEVD-CHO corrected values were exported as Microsoft Excel files and analysed.

### In vivo assays

NOD/SCID gamma (NOD.Cg-Prkdcscid Il2rgtm1Wjl/SzJ) (NSG) mice were bred in-house. Approval by the University’s animal care and ethics committee was obtained (18-MDM-01) and in vivo experiments were performed according to applicable laws and regulations. Single A375X1 resistant cells (2*10^6^ cells) were resuspended in 100 μL of 1:1 mixed serum-free medium and matrigel (BD Biosciences) and injected subcutaneously (right and left flank) of 6–8 week-old mice. Mice were randomized at day 10 (*n* = 5, tumour volume around 100mm^3^), and daily oral treatment was started for 7 consecutive days with vehicle, 45 mg/kg vemurafenib, 50 mg/kg ceritinib, or the combination of ceritinib and verumafenib. Drugs were formulated in 4% DMSO, 30% PEG 300, 5% Tween 80, ddH2O. Tumour growth was followed and tumour volume was calculated by the formula LxW^2^/2.

### Patient samples and immunohistochemistry

Tumour samples were collected from melanoma patients at the Klinikum Dortmund (in Germany). Samples were obtained with patient consent and approval of the ethic committee (Ethikkommission der Ärztekammer Westfalen-Lippe und der Westfälischen Wilhemls-Universität, reference number 2015–178-f-S). Patient studies were conducted according to the Declaration of Helsinki and the Belmont Report.

Immunohistochemistry on formalin-fixed paraffin-embedded (FFPE) slides from melanoma samples was performed at the Integrated Biobank of Luxembourg (IBBL). Additional information is included in Additional file 2: Supplementary Methods.

### Extracellular vesicles isolation and labelling

Donor cells (both A375 and A375X1) were slowly adapted to serum-free medium (UltraCulture, Lonza BioWhittaker). Culture supernatants (100 ml) were harvested, centrifuged 2 × 10 minutes at 400 g, followed by 20 min at 2000 g to remove cells and cell debris. Extracellular vesicles were isolated by ultracentrifugation (70 min at 110000 g, 4 °C) by using a MLA-55 fixed rotor followed by flotation on an Optiprep cushion (Axis-Shield, 17%) for 75 min at 100000 g at 4 °C using a swinging MLS-50 rotor. After a PBS wash (110000 g, 70 min), extracellular vesicles were resuspended in PBS and frozen at − 80 °C. Protein quantification was performed using Pierce™ BCA Protein Assay Kit (Termo Fisher) according to the manufacturer’s instructions.

To label extracellular vesicles, culture supernatants were processed as mentioned above. After ultracentrifugation at 110000 g, the pellet was resuspended in 250 μl of PBS and stained with 5 μl of PKH67 (Sigma) for 30 min at 37 °C. To remove excess dye, this suspension was loaded on the Optiprep cushion, followed by a PBS washing step. 10 μg of labelled EVs were added to the cells; after 24 h cells were fixed and stained with SiR-actin kit (Spirochrome).

### Visualization of EVs

For electron microscopy, a drop of extracellular vesicles suspended in PBS was deposited on Formvar-carbon-coated electron microscopy grids. The samples were fixed with 2% PFA, labelled with anti-CD63 (Abcam) and immunogold-labelled using protein A coupled to 10 nM gold (PAG10) as previously described [18].

### EV mass spectrometry

A liquid chromatography-tandem mass spectrometry (LCMS/MS) system was used to study the protein composition of EVs. The detailed protocol is shown in the Additional file 2: Supplementary Methods.

### EV transfer experiments

For the transfer assays, 25000 cells in 24 well plates were seeded in RPMI medium. The day after, following 1 h pre-treatment with 1 μM of PLX4032, increasing concentrations of resistant EVs were added to the cells. After 7 h, cells were collected for western blot analysis.

### Immunofluorescence

For ALK immunofluorescence, A375 or A375X1 cells grown on glass coverslips were treated with 10 μg of EVs for 24 h. Cells were washed with PBS and fixed with 4% paraformaldehyde in PBS for 10 min at room temperature. The coverslips were washed three times in PBS-Tween (0.05% Tween 20). Then, cells were permeabilised with PBS 0.5% Triton X-100 for 10 min at room temperature, and blocked in PBS plus 2% bovine serum albumin (BSA) for 15 min. Cells were incubated with ALK antibody, diluted in PBS plus 2% BSA, for 1 h at room temperature. Coverslips were washed 3 times with PBS and treated with Alexa Fluor 488 donkey anti-rabbit IgG (Invitrogen) for 1 h at room temperature. Coverslips were washed and mounted with Gold antifade reagent with DAPI (Invitrogen). The cells were visualised by Andor Revolution Spinning Disk confocal microscopy, mounted on a Nikon Ti microscope (60× oil objective) and the images were analysed with ImageJ software.

### Statistical analysis

Statistical analysis was performed with the GraphPad Prism software (version 5). All data are presented as mean of three biological replicates ± s.d. and were analysed either with paired Student’s t-test or one-way ANOVA coupled with Tukey’s multiple comparison tests. Differences in tumour volumes among groups of treated mice were tested using a two-way ANOVA (treatment factor *p* = 0.0004) followed by multiple comparison t-tests corrected with the Holm-Sidak method; data are presented as mean tumour volumes (mm^3^) ± SEM. Tumour weights were analysed by unpaired student’s t-tests with Welch’s correction at end-point conditions and represented as mean tumour weights (mg) ± SEM.

### Supplemental information

Supplemental information includes Additional file 2: Supplementary methods, Additional file 1: Tables S1 and S2 and Additional file 3: Figures S1–S7.

## Results

### Characterisation of vemurafenib-sensitive and -resistant A375 melanoma cells

**Fig. 1:**
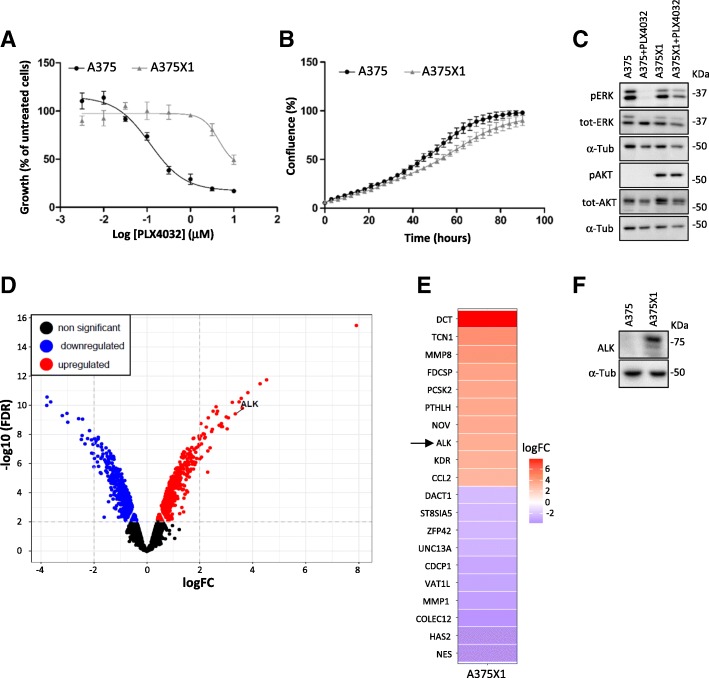
Characterisation of resistant melanoma cells. (**a**) Vemurafenib dose-response analysis in sensitive A375 (black) and resistant A375X1 cells (grey). (**b**) Growth comparison between untreated sensitive cells versus resistant cells under constant PLX4032 treatment (1 μM). (**c**) Western blot analysis of A375 and A375X1 in absence or presence of PLX4032 (3 h). Before PLX4032 treatment cells were starved for 16 h. α-Tubulin was used as a loading control; representative blots of three biological replicates are shown. (**d**) Vulcano plot showing differentially expressed genes in resistant compared to sensitive melanoma cells (FDR < 0.01, at least 1.5-log fold change). (**e**) Top differentially expressed mRNAs in resistant cells. (**f**) Western blot analysis detecting ALK^RES^ only in resistant A375X1 cells. α-Tubulin was used as a loading control; representative blots of three biological replicates are shown

BRAF^V600E^ A375 cells were made resistant to 1 μM PLX4032 over a period of eight weeks with constant exposure to the drug. Twenty different resistant clones were isolated in order to investigate new mechanisms of resistance. The fastest growing clone under PLX4032 treatment, named A375X1, was selected for further experiments. The resistance of the established cell clone was examined by dose-responses analysis (Fig. 1a) and by growth assays (Fig. 1b) showing that resistant cells have similar growth rates under PLX4032 compared to untreated parental cells. The resistant clone showed reactivation of the MEK1/ERK1/2 pathway compared to parental cells treated with BRAF inhibitor, as shown by the phosphorylation of ERK1/2. In addition, the resistant cells also displayed increased pAKT levels suggesting an activation of the Pi3K/AKT pathway (Fig. 1c).

To elucidate underlying mechanisms of resistance, we first performed gene expression analysis on drug-sensitive and -resistant A375 cells. Differentially expressed candidates emerging in the resistant cells (FDR < 0.01, at least 1.5-log fold change) were plotted (Fig. 1d). In accordance with our previous data [15], several genes were upregulated in the resistant A375X1 clone such as the Proprotein Convertase Subtilisin/Kexin type 2 (PCSK2), the Dopachrome Tautomerase (DCT), the Matrix Metallopeptidase 8 (MMP8) (Fig. 1e). Additionally, Anaplastic Lymphoma Kinase (ALK) was also identified in the top differentiated genes. As ALK has recently been described to be present in an oncogenic form in melanoma patients [14], we focused our attention on ALK.

### Characterisation of ALK

ALK is known to be rearranged or mutated in several malignancies [13]. ALK protein could be detected by western blot (Fig. 1f), although the detected band was smaller (multiple bands around 75 KDa) than expected for full length ALK (200 KDa). In the wake of the discovery of the novel ALK isoform (ALK^ATI^) identified in melanoma patients, we next characterised ALK^RES^ protein by performing 5′-rapid amplification of cDNA ends (5′-RACE) followed by Sanger sequencing. Results identified a truncated ALK starting from exon 18 (Additional file 3: Figure S1) fused to a sequence aligning to murine leukemia virus (MMLV). ALK was additionally fully sequenced confirming the presence of a protein coding sequence starting from exon 18 to exon 29 (Additional file 3: Figure S2). PCR amplification, using primers located in the kinase domain of ALK, confirmed the presence of ALK in our resistant cells and in EML4-ALK positive lung cancer cells, which served as a positive control (Additional file 3: Figure S3A). The amplification of this unusual MMLV-ALK fusion gene using primers at the interface between MMLV and ALK, was exclusively observed in the drug resistant A375X1 clone (Additional file 3: Figure S3B). Next, seven melanoma cell lines and normal melanocytes were screened for the presence of ALK transcripts using primers in the kinase domain of ALK but none except A375X1 were positive for ALK (Additional file 3: Figure S3C). Considering the mRNA sequence, ALK^RES^ contains 70 extracellular amino acids, the transmembrane domain and the whole cytoplasmic domain. To further analyse the protein, immunoprecipitation was performed (Additional file 3: Figure S3D), revealing mainly two bands on western blot. Mass spectrometry confirmed that both bands correspond to ALK (Additional file 3: Figure S4) and that no viral protein sequence was fused to ALK^RES^. Taken together, these data suggest the existence of an unusual fusion between the C-terminus of ALK and a MMLV sequence at the mRNA level but not at the protein level, giving rise to a novel ALK isoform (ALK^RES^).

Interestingly, the upper band (in western blot) showed a higher mass than expected from the sequence, while the lower one had the expected molecular weight. Due to the presence of 70 extracellular amino acids in the sequence, we suspected ALK^RES^ to be glycosylated, which would explain the shift observed in the western blot. Treatment of the immunoprecipitated ALK^RES^ with glycosidases (EndoH and PNGaseF) led to a size shift especially upon PNGaseF treatment (Additional file 3: Figure S3D). This indicates the presence of a complex glycosylation which suggest a protein localization in the Golgi and/or at the plasma membrane. Using immunofluorescence, we detected ALK mostly in perinuclear structures, which resemble the Golgi and to a lesser degree in the plasma membrane with weaker diffused intracellular staining (Additional file 3: Figure S3E). Taken together, these data confirm the presence of a truncated transmembrane ALK protein, which is different from the recently identified ALK^ATI^ in melanoma and from previously described fusion proteins (Additional file 3: Figure S3F).

### ALK confers acquired resistance to melanoma cells

**Fig. 2:**
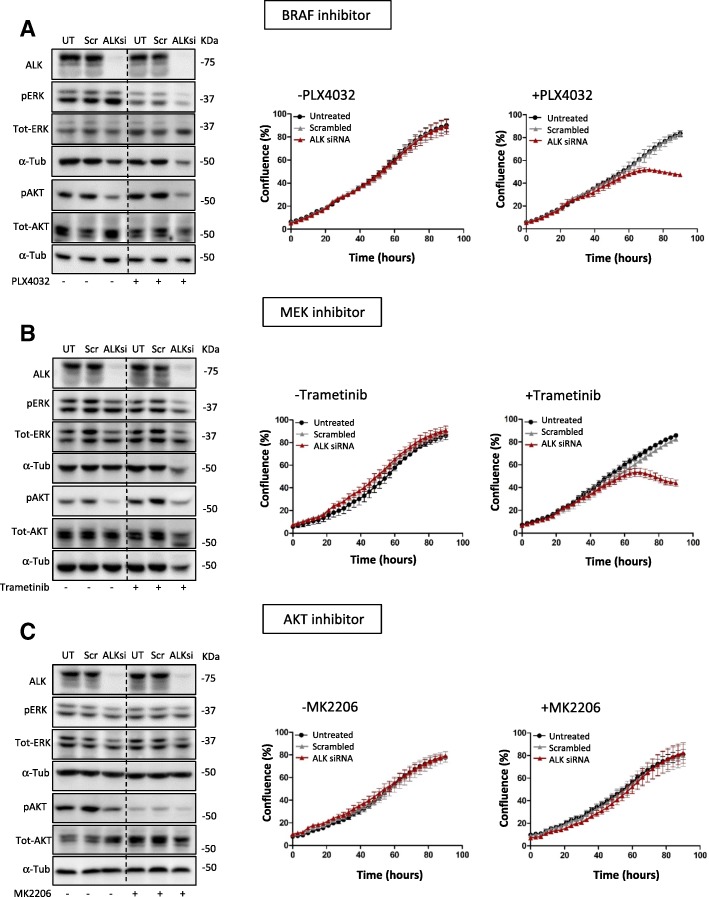
Knock down of ALK^RES^ re-sensitises resistant cells to BRAF inhibition. A375X1 cells were transfected with three different siRNAs against ALK or a scrambled control (100 nM) for 72 h. 48 h prior to collection, the cells were incubated with either PLX4032 (1 μM) (**a**) or Trametinib (3 nM) (**b**) or MK2206 (1 μM) (**c**). α-Tubulin was used as a loading control and one representative of three biological replicates is shown. (**a**-**c**) Corresponding growth assays on the right. The plates were imaged every 3 h using an IncuCyte ZOOM live cell microscope (Essen BioScience) and images were taken for a total of 90 h. Results are shown for one representative of three biological replicates

Many mechanisms of melanoma drug resistance have recently been put forward [19] but so far, ALK has not been implicated. Therefore, we investigated the involvement of ALK in mediating drug resistance by knocking down ALK using an siRNA approach. Western blot analysis and growth assays were performed in cells treated with both scrambled and ALK siRNA in the absence or presence of BRAF, MEK and AKT inhibitor (Fig. 2a, b and c, respectively).

A strong reduction of ALK^RES^ expression levels following the siRNA treatment was observed (Fig. 2). Following the down regulation of ALK^RES^, a decrease in pERK was detected in presence of PLX4032 while no change was observed in absence of the drug, which was expected since BRAF^V600E^ is not inhibited and activates the ERK1/2 pathway. In addition, lower levels of pAKT were detected under both conditions (Fig. 2a). No change in growth behaviour was observed in the absence of PLX4032 whereas growth inhibition was detected when cells were treated with ALK siRNA in combination with PLX4032 (Fig. 2a). Similar results were obtained when cells were treated with a MEK inhibitor (Fig. 2b). To assess the importance of the AKT pathway, cells were additionally treated with a combination of ALK siRNA and the AKT inhibitor (MK2206). As expected, although pAKT was reduced when cells were treated with both siRNA alone and MK2206 (Fig. 2c), no effects were observed on cellular growth (Fig. 2c). Altogether, these results indicate that ALK^RES^ is mediating acquired resistance by activating the MAPK pathway. In the absence of ALK^RES^, resistant melanoma cells respond again to both BRAF and MEK inhibitors.

### Combination of ALK inhibitors with vemurafenib efficiently inhibits cell growth and leads to increased apoptosis in resistant melanoma cells

**Fig. 3:**
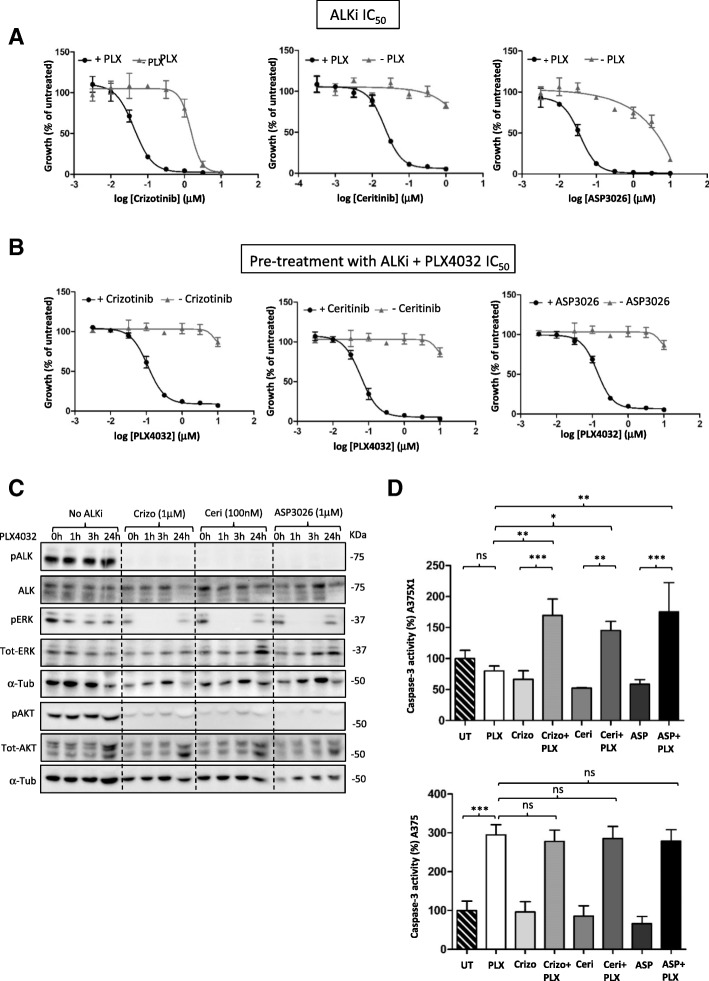
The combination of ALK and PLX4032 inhibitors is efficient in resistant melanoma cells. (**a**) ALK inhibitors (Crizotinib, Ceritinib and ASP3026) dose-response in resistant A375X1 cells cultured in the absence or presence of 1 μM of PLX4032. (**b**) PLX4032 dose-response in resistant cells cultured with or without 1 μM of ALK inhibitors. (**c**) Western blot analysis of resistant A375X1 cells treated with PLX4032 for the indicated time points in the presence of absence of ALK inhibitors. α-Tubulin was used as a loading control and one representative of three biological replicates is shown. (**d**) Apoptosis assays showing the activity of caspase-3 in resistant and sensitive cells treated either with single inhibitors or with a combination of ALK and BRAF inhibitors, normalised to the untreated control. Error bars represent the standard deviation of three technical replicates of three biological replicates. Statistical significance was determined with a one-way ANOVA coupled with Tukey’s multiple comparison tests. *p < 0.05, **p < 0.01, ***p < 0.001

Next, we asked whether the dependence of A375X1 melanoma cells on ALK could be exploited to overcome BRAF inhibitor resistance and we treated the cells with three different ALK inhibitors (Crizotinib, Ceritinib and ASP3026) alone or in combination with PLX4032. Dose-response analysis showed that ALK inhibitors combined with the BRAF inhibitor were much more efficient in suppressing cellular proliferation compared to single treatments (Fig. 3a). In addition and importantly, pre-treatment of resistant cells with 1 μM of ALK inhibitors restored sensitivity to PLX4032 (Fig. 3b). Western blot analysis showed that the three ALK inhibitors alone inhibited ALK^RES^ phosphorylation and the downstream pAKT signalling, while only when combined with vemurafenib, pERK was additionally inhibited (Fig. 3c).

To examine whether the combination of inhibitors was exclusively inhibiting growth or whether it could also induce cell death of resistant melanoma cells, apoptosis assays were performed in both resistant and sensitive cells. As expected, apoptosis was not detected when resistant cells were treated either with PLX4032 alone or with one of the three ALK inhibitors. However, combination treatment with both types of inhibitors induced a significant increase in apoptosis (Fig. 3d). As for the sensitive cells, apoptosis was induced exclusively when the cells were in presence of PLX4032 and additional ALK inhibitors did not increase the level of apoptosis induced by PLX4032 (Fig. 3d).

### ALK detection in melanoma patient samples and ALK inhibition in vivo

**Fig. 4:**
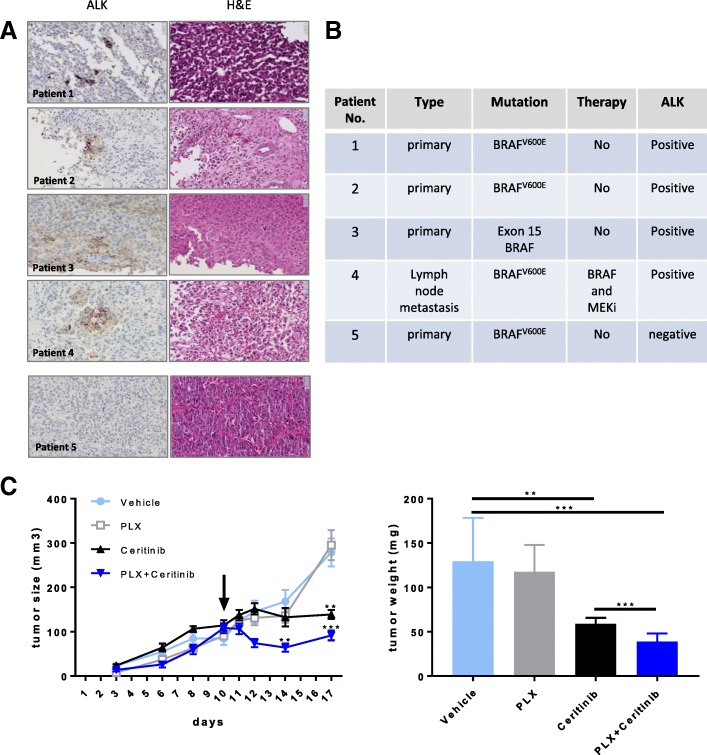
ALK is detected in melanoma samples. (**a**) Immunohistochemistry and corresponding Hematoxylin and Eosin staining of FFPE slides of melanoma patient samples. ALK immunohistochemistry reveals a minor population of moderate immunopositive cells scattered throughout the tumour (Patients 1–4). Patient 5 is representative for ALK-negative sample. Magnification: 40X. (**b**) Table summarizing patient information. (**c**) Combination treatments with BRAF and ALK inhibitors strongly reduce melanoma tumour volumes. NSG mice were injected subcutaneously with 2 million A375-X1 cells. After 10 days, treatment was initiated by daily gavage (arrow). Tumour growth was followed over time (left panel) and weight of extracted tumours were measured (right panel). Data are presented as means of tumour volumes (mm^3^) ± SEM and means of tumour weights (mg) ± SEM, *p < 0.05, **p < 0.01, compared to vehicle-treated tumours (left panel); ***p < 0.001 between groups as indicated (right panel)

To broaden the scope of our findings and to better understand if the proposed drug combination could be of clinical relevance for patients, we examined the presence of ALK in 26 FFPE samples derived from both primary and metastatic melanoma patients. Positive labelling was identified in 4 out of 26 cases (corresponding to 15%). Generally, only a minority of tumour cells was ALK-positive and those positive cells tended to be scattered randomly throughout the tumour as isolated cells or small clusters (Fig. 4a). The general staining intensity was moderate. Among the ALK-positive samples, 3 were primary tumours sampled before treatment. In particular, patient 1 and 2 were BRAF^V600E^ positive while patient 3 carried an inactivating mutation in exon 15 of the BRAF gene. Patient 4 represents a lymph node metastasis positive for BRAF^V600E^, sampled after the development of drug resistance to BRAF/MEK inhibitors (Fig. 4b). Even within this small cohort, the data suggest that pharmacological inhibition of ALK combined with BRAF inhibitors might represent an interesting therapeutic opportunity for a subset of melanoma patients.

As previously mentioned, a truncated form of ALK was recently identified in 11% of melanoma patients as well as other somatic mutations able to activate ALK [13, 14]. To determine how many patients could potentially benefit from dual inhibition of BRAF and ALK, we analysed the TCGA database focusing on melanoma patients. Of 470 entries, 203 patients have a BRAF^V600^ mutation, 111 patients have mutations in ALK and 41 have both BRAF^V600^ and ALK. Of these 41 patients, 14 were found to have BRAF^V600^ coupled with missense mutations in ALK (Additional file 3: Figure S5). Our data together with the TCGA data suggest that the combination of BRAF and ALK inhibitors could be a promising strategy to overcome drug resistance in a group of patients carrying both BRAF^V600^ and expressing ALK.

To assess the effect of BRAF and ALK inhibition in vivo, we tested vemurafenib, ceritinib and the combination of both in mice harbouring A375-X1-induced melanoma tumours (Fig. 4c). The combined inhibition of BRAF and ALK stopped tumour growth, supporting the clinical relevance of our findings.

### Characterisation of EVs secreted from vemurafenib-sensitive and -resistant A375 melanoma cells

EV-mediated intercellular communication has recently been described as an important mechanism to propagate drug resistance [20]. To investigate such a potential transfer of drug resistance in our model, EVs were isolated from A375 parental and A375X1 resistant cell supernatants. The purity of isolated EVs was assessed by western blot analysis to detect the presence of generic and well known EV markers. As expected, CD9, CD81 were enriched in EV preparations while TSG101 was found in both cells and EVs (Additional file 3: Figure S6A). Electron microscopic visualization of EVs revealed their characteristic and artificial cup-shaped morphology. Furthermore, immunogold labelling was positive for CD63 (Additional file 3: Figure S6B). To study vesicle uptake by melanoma cells, purified EVs from resistant cells were labelled with a green fluorescent dye (PKH67) and incubated with sensitive A375 melanoma cells for 24 h showing that sensitive A375 take up resistant EVs (Additional file 3: Figure S6C).

### Drug resistance can be transmitted by EVs

**Fig. 5:**
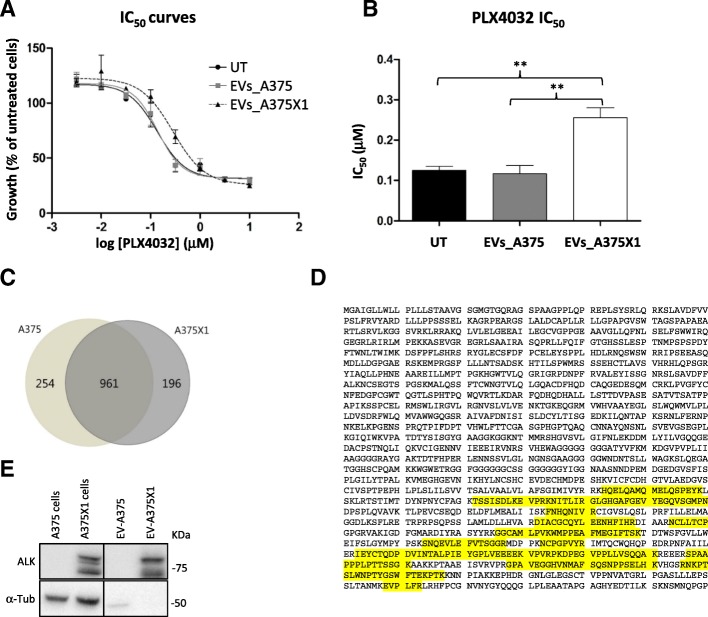
EVs can transfer functional properties. (**a**) Sensitive A375 melanoma cells were co-cultured with both EV-A375 and EV-A375X1 (10 μg/ml). After 24 h, vemurafenib dose-response analysis was performed to calculate the IC_50_. Representative dose-response curves of sensitive A375 (black), sensitive A375 plus EV-A375 (grey) and sensitive A375 plus EV-A375X1 (dotted line). (**b**) PLX4032 IC_50_ values of sensitive A375 (black), sensitive A375 plus EV-A375 (grey) and sensitive A375 plus EV-A375X1 (white). Error bars represent the standard deviation of three biological replicates. Statistical significance was determined using paired Student’s *t*-tests. *p < 0.05, **p < 0.01, ***p < 0.001. (**c**) Venn diagram showing unique and shared proteins identified by mass spectrometry in EVs isolated from both sensitive A375 and resistant A375X1 cells. (**d**) ALK consensus sequence in which the highlighted peptides are the ones detected by MS in the resistant EVs. (**e**) ALK western blot analysis of sensitive and resistant cells and corresponding EVs. Results are shown for one representative of three biological replicates

To study the capability of EVs isolated from resistant cells to transfer the acquired drug resistance to sensitive cells, we first determined the dose response to PLX4032 following EV uptake (Fig. 5). 50% cell growth inhibition (IC_50_) was calculated to assess differences in drug response between A375 cells, A375 cells pre-incubated with EVs isolated from the same A375 cells or pre-incubated with EVs isolated from resistant A375X1 cells. No significant difference was observed when sensitive cells were incubated with their own EVs while significantly higher IC_50_ were scored when cells were incubated with resistant-EVs (Fig. 5a and b), indicating that the uptake of resistant EVs renders the sensitive cells more resistant to PLX4032. These findings suggest that the drug resistance phenotype can be transferred by EVs.

### ALK^RES^ is present in resistant EVs and can be transferred

Next, we characterised the protein content of EVs to identify potential players involved in transferring drug resistance. Proteomic analysis of sensitive and resistant EVs, isolated from the supernatants of the corresponding cell lines identified about 1400 proteins. Of these, 962 were common in both, 254 were unique for sensitive EVs and 196 were unique for resistant EVs (Fig. 5c). Interestingly, ALK^RES^ was again exclusively detected in the resistant EVs, whose sequence coverage clearly suggests a truncated protein as peptides were only detected in the C-terminal part of the protein (Fig. 5d). We confirmed the presence of ALK^RES^ in both resistant cells and in the corresponding EVs while it was not detectable in sensitive cells and their EVs (Fig. 5e).

**Fig. 6:**
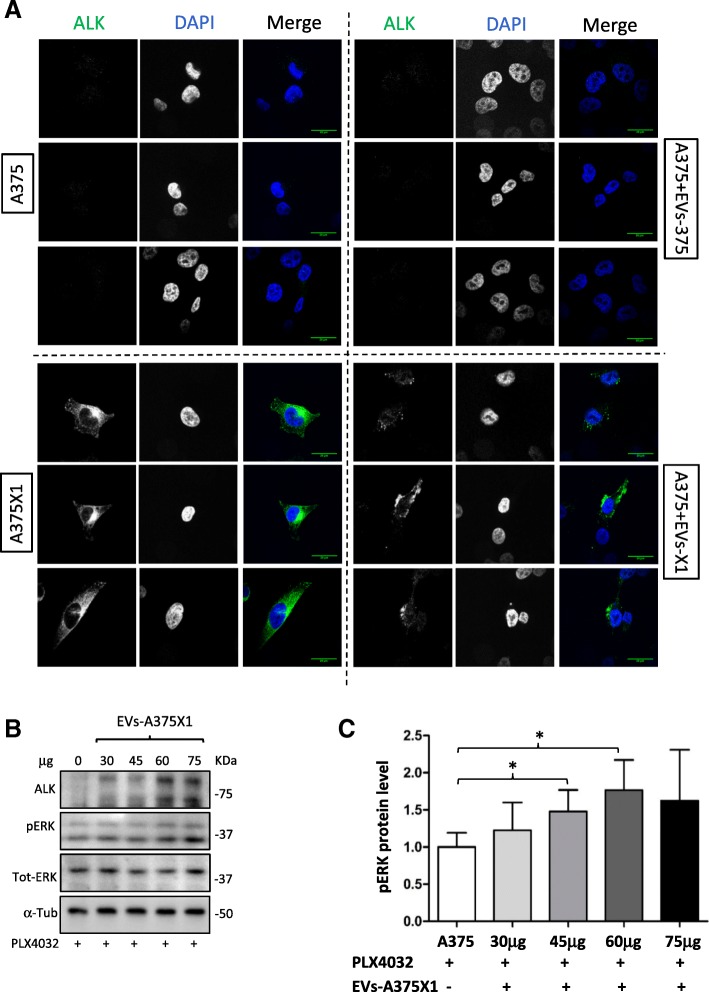
Functional ALK^RES^ is transferred to sensitive cells via EVs. (**a**) Sensitive A375 melanoma cells were co-cultured with 10 μg of both EV-A375 and EV-A375X1. After 24 h, untreated A375 cells, resistant A375X1 cells and A375 co-cultured with both types of EVs were fixed and stained for ALK. Images were captured by fluorescence confocal microscopy. Representative images of two biological replicates. Scale bar, 20 μm. Blue: nucleus; green: ALK. (**b**) Sensitive A375 cells were treated with 1 μM of PLX4032. After 1 h, increasing concentrations of resistant EVs were added to the cells for additional 6 h. α-Tubulin was used as a loading control; representative blots of three biological replicates are shown. (**c**) Quantification of pERK levels, normalised to the untreated control. Error bars represent the standard deviation of three biological replicates. Statistical significance was determined using paired Student’s t-tests. *p < 0.05, **p < 0.01, ***p < 0.001

EVs are known to promote horizontal transfer of different molecules to recipient cells [21]. However, the transfer of phenotypic traits and functional properties by EVs and their content is often difficult to establish due to the long and multistep isolation protocol and further limited by recovery amounts. To further investigate the role of ALK^RES^ in mediating drug resistance, we asked whether ALK^RES^ could be transferred through EVs and remain functional in recipient cells. Confocal microscopy of immunofluorescence staining for ALK^RES^ showed its presence in sensitive cells after 24 h of exposure to resistant EVs (Fig. 6a) suggesting the successful transfer of ALK^RES^ between cells. Of note, staining for ALK was not restricted to punctuate structures but was present in the cytoplasm suggesting the diffusion of ALK^RES^ from endocytic compartments that have taken up the EVs to the rest of the cell. Finally, and to examine whether transferred ALK is functional, we analysed whether the addition of ALK-containing resistant-EVs could activate the MAPK pathway. Sensitive A375 melanoma cells were initially treated with 1 μM of PLX4032 to reduce their basal level of pERK. Next, increasing concentration of resistant EVs were added to the cells for 6 h. Levels of pERK increased in accordance with increased concentration of EVs (Fig. 6b and c) suggesting an activation of ERK by EV-transferred ALK^RES^ (Additional file 3: Figure S7). This small but reproducible augmentation of pERK signals was astonishing given the probably minute amounts of active protein transported by EVs.

## Discussion

Over the past few years, the implementation of accurate screening programs together with major advances in treatment choices have vastly improved the life expectancy for advanced stage melanoma patients [22]. The availability of specific inhibitors targeting mutated BRAF and the downstream MAPK signalling pathway or other kinases activated in melanoma, together with immunotherapies that de-block inhibition of T cell responses against the tumour, offer potent ways to fight this cancer [23]. However, immunotherapies are only successful in less than 30% of cancer patients, often have severe side effects, lead to resistance and are still very costly [22, 24, 25]. On the other hand, treatment of BRAF-mutant melanoma patients with BRAF inhibitors in monotherapy or in combination with MEK inhibitors is limited by both acquired and intrinsic drug resistance [11]. The re-activation of the MAPK signalling pathway due to secondary mutations is one of the key mechanisms driving acquired resistance to BRAF inhibitors. Promising new drugs such as compounds inducing ER stress, targeting mitochondria biogenesis or metabolic pathways (PDKi) that are effective in both intrinsically and acquired resistant cells and/or xenografts have recently been postulated as potential candidates for second line treatments [17, 26–28]. A deeper understanding of the re-activation mechanisms of the MAPK pathway will aid the selection of appropriate novel therapies to improve survival of melanoma patients.

In this study, we report ALK to be involved in driving resistance in a subclone of BRAF-resistant cells. Several translocations, mutations or amplifications render ALK oncogenic in different cancer types [13]. So far, 22 different genes have been described to fuse with the C-terminal part of ALK making the ALK locus particularly prone to activating translocations [13]. The various translocations normally produce constitutively activated ALK fusion proteins, which can signal through the MAPK signalling pathway, the PI3K/AKT pathway or the JAK/STAT pathway contributing to cell proliferation and survival [12]. Therefore, ALK fusion proteins are already important clinical targets in non-small cell lung cancer (EMLA4-ALK) but have also been described in diffuse large cell lymphoma (NPM-ALK) and in inflammatory myofibroblastic tumour (TPM3-ALK). In addition, a new ALK transcript consisting of a fragment of intron 19 followed by exons 20–29 that resulted from an alternative transcription initiation was recently identified in 11% of melanoma patients [14]. In our study, an activating translocation with a murine leukemia viral sequence was observed, which leads to a truncated protein lacking the N-terminal part (exons 1–17). We confirmed by whole genome sequencing that this MMLV was stably inserted in our A375 cells (data not shown). The identification of MMLV has been reported for many cancer cell lines, including melanoma, across several laboratories [29, 30] suggesting MMLV as a regular resident in cancer cells. Nevertheless, the activation of ALK by a murine retrovirus suggests that other sequences from human retroviruses or their closely related human retrotransposons or any other translocating sequence can activate this oncogene in humans.

Most of the ALK variants described so far (overexpressed wild-type ALK, EML4-ALK, NPM-ALK, ALK^ATI^, ALK^R1275Q^, ALK^F1174L^) were shown to trigger proliferation and tumourigenesis and to be sensitive to ALK inhibitors [14, 31–34]. In this context, a phase 2 clinical trial has been launched to test the effect of ALK inhibitor in melanoma patients harboring ALK alterations or aberrant ALK expression (https://clinicaltrials.gov/ct2/show/NCT03420508#studydesc).

In our study, to determine therapeutic responses, we tested three different ALK inhibitors in combination with BRAF inhibitor. As expected, both knock down and inhibition of ALK^RES^ did not have any effect per se on the growth of resistant cells as phosphorylation of ERK was not inhibited. Only with the combination of BRAF inhibition (and subsequently ERK), cell growth was suppressed and apoptosis induced. This demonstrates that ALK^RES^ modulates sensitivity to BRAF inhibition. The combined inhibition of BRAF and ALK could therefore be of immediate clinical relevance to those patients who acquired secondary mutations within ALK or for those who carry BRAF^V600E^ together with an oncogenic isoform of ALK and show intrinsic resistance to BRAF inhibitor monotherapy.

Importantly, the presence of ALK^RES^ in resistant cells was mirrored in the corresponding EVs, suggesting that circulating vesicles might be useful diagnostic tools to identify biomarkers of resistance. The detection of ALK^RES^ in EVs prompted us to examine whether this new oncogenic protein could also be transferred to other melanoma cells. The transfer of phenotypic traits through EVs is an emerging field of research [35, 36]. Here, we describe for the first time a functional transfer of a truncated kinase (ALK^RES^) by EVs likely involved in the propagation of a drug resistance phenotype in melanoma. Of note, the modest effect induced by resistant-EVs (Fig. 5a and b, Fig. 6b and c) is not surprising: EV preparations represent an heterogeneous mixture of vesicles [37] and if only a subtype of EVs carries ALK, its efficacy will be diluted by the presence of other types of EVs, which also transport a spectrum of different proteins and small RNAs [21, 37]. Furthermore, the isolation protocol might affect the real biological activities of EVs. In addition, it is important to note that ALK might not be the only mediator of drug resistance dissemination and that several players are likely working together to contribute to this phenotype.

## Conclusion

To achieve more effective and personalised second line treatments for melanoma and other cancer patients, understanding the individual mechanisms of drug resistance is crucial. Our findings describe a novel mechanism driving the acquisition and spreading of a drug resistant phenotype in melanoma. To the best of our knowledge, this is the first study demonstrating i) the expression and involvement of a novel truncated ALK protein (ALK^RES^) in drug-resistance, ii) that the inhibition of ALK restores sensitivity to BRAF inhibitors; iii) the presence of functional ALK^RES^ within EVs, which likely mediates the transfer of drug resistance and iv) that the combined inhibition of BRAF and ALK is a promising clinical treatment option for certain melanoma patients.

## Additional files

Additional file 1: **Table S1.** List of Primer sequences. **Table S2.** ALK siRNA sequences. (PDF 288 kb)

Additional file 2: Supplementary methods. (PDF 269 kb)

Additional file 3: Supplementary **Figures S1–S7.** (ZIP 3175 kb)

## Abbreviations

ALK: Anaplastic Lymphoma Kinase
ALKi: ALK inhibitors
BRAF: B-Rapidly Accelerated Fibrosarcoma
EML4: Echinoderm Microtubule Associated Protein Like 4
ERK: Extracellular-Signal Regulated Kinase
EV: Extracellular Vesicle
FDA: Food and Drug Administration
FFPE: formalin-fixed paraffin-embedded
IP: immunoprecipitation
LDL: Low-density lipoprotein domain
MAM: meprin, A-5 protein, and receptor protein-tyrosine phosphatase mu
MAPK: Mitogen-Activated Protein Kinase
MS: Mass Spectrometry.
NPM: Nucleophosmin.
TCGA: The Cancer Genome Atlas.
