# Supplementary material for: A new ALK isoform transported by extracellular vesicles confers drug resistance to melanoma cells
Source: Mol Cancer. 2018 Oct 5;17:145. doi: 10.1186/s12943-018-0886-x (PMC6172729; doi:10.1186/s12943-018-0886-x)
Supplement: Supplementary file 1 — Table S1. List of Primer sequences. Table S2. ALK siRNA sequences. (PDF 288 kb) [file 12943_2018_886_MOESM1_ESM.pdf]

Table S1: List of Primer sequences

Gene	Forward	Reverse	Application
ALK	5’ AGGGGGCTTGGGTCGTTGGGCATT 3’	5’ TGTCTCGGTGGATGAAGTGGTTTTCC 3’	5’RACE
ALK	5’ TGATGGAAGGCCACGGG 3’	5’ TCAGGCAGCGTCTTCACA 3’	Sequencing
ALK	5’ CCTCATTCGGGGTCTGG 3’	5’ CCCTTTCTATAGTAGCTCGCC 3’	Sequencing
ALK	5’ AACTGCCTCTTGACCTGTCC 3’	5’ TTTTGCCTGTTGAGAGACCA 3’	Sequencing
ALK	5’ GGAAGAGAAAGTGCCTGTGAG 3’	5’ AAGAGAAGTGAGTGTGCGACC 3’	Sequencing
ALK	5’ GGGGAGGTGTATGAAGGC 3’	5’ CGGTGGATGAAGTGGTTTT 3’	PCR
MMLV-ALK	5’ CAGGCAGTGATGGAAGGC 3’	5’ CGGTGGATGAAGTGGTTTT 3’	PCR
ALK	5’ GCATTGTGTCACCCACCC 3’	5’ CATGGCTTGCAGCTCCTG 3’	qPCR

Table S2: ALK siRNA sequences

siRNA1	GGGCCUGUAUACCGGAUAA
siRNA2	GUGCCAUGCUGCCAGUUAA
siRNA3	CCGCUUUGCCGAUAGAAUA
