# Supplementary material for: A new ALK isoform transported by extracellular vesicles confers drug resistance to melanoma cells
Source: Mol Cancer. 2018 Oct 5;17:145. doi: 10.1186/s12943-018-0886-x (PMC6172729; doi:10.1186/s12943-018-0886-x)
Supplement: Supplementary file 2 — Supplementary methods. (PDF 269 kb) [file 12943_2018_886_MOESM2_ESM.pdf]

Supplementary methods

Immunohistochemistry

The staining was performed on an automated Benchmark XT device (Ventana, Tucson, AZ, USA) using the CC1 antigen retrieval protocol (Cell conditioning 1 buffer, basic pH, 92 minutes). Primary antibody against ALK (D5F5) was used at a 1:100 dilution, with an incubation time of 30 minutes. The secondary antibody was retrieved from an OptiView DAB IHC detection kit + Amplification (Ventana). Appropriate positive and negative controls were used.

EV mass spectrometry

Analysis of the EV proteome

EV samples containing 100µg of protein were reduced with 10mM DTT in the presence of 1% sodium deoxycholate (SDC) (100mM Tris buffer, pH8) for 1 hour at 37°C. After reduction, proteins were alkylated with 25mM iodoacetamide for 1 hour at 37°C, in the dark, until quenched by 10mM N-acetyl cysteine for 30 minutes, at room temperature. Then, protein extraction was performed using a methanol/chloroform precipitation method. Briefly, 150µl of EV sample were mixed with 1ml of a methanol:chloroform:water (2:1:2) solution, and vortexed before centrifugation for 5 minutes, at 5000g. The upper layer of the solution was removed and 600µl methanol were added. After vortexing followed by centrifugation for 30 minutes at 20000g, the supernatant was removed to recover the protein pellet. The air-dried pellet was reconstituted for trypsin digestion in 1% SDC (100mM Tris buffer, pH 8.8) for 16 hours, at 37°C. After trypsin digestion, SDC was precipitated by acidification using 1% formic acid (FA). The white pellet formed after acidification was removed by centrifugation, for 20 minutes at 20000g. The supernatant was cleaned up using a C18 cartridge (Sep-Pak®, 1cc, Waters Corporation) and dried in a speedvac concentrator (Savant SPD 111V, Thermo Fischer Scientific). The dried peptide mixture was reconstituted with 100 µl of 0.1% FA / 5% acetonitrile (ACN) for LC-MS analysis.

LC-MS/MS analysis

The reconstituted sample was further diluted (5 times) and mixed with 7.5fmol of a peptide-retention-time calibration mixture (Thermo Fisher Scientific) for retention time control of the data. Peptides were separated with a reverse-phase liquid chromatography (LC) system using

an Ultimate 3000 RSLCnano (Thermo Fisher Scientific) equipped with an Acclaim PepMap RSLC column (15 cm × 75 µm, C18, 2 µm, 100 Å) (Thermo Fisher Scientific). 1µl of each sample was used for proteomic analysis. Peptide elution was performed by applying a mixture of solvents A and B to the LC system. Solvent A was an aqueous solution with 0.1% FA, and solvent B was ACN with 0.1% FA. A linear gradient of 2–35% solvent B at 300nL/min was applied over 48 minutes followed by a washing step (5 minutes at 90% solvent B) and an equilibration step (10 minutes at 2% solvent B). Mass spectrometry analysis was performed using a Q-Exactive Plus mass spectrometer (Thermo Scientific, Bremen, Germany) equipped with a nano-electrospray source. For ionization, uncoated SilicaTips (12cm, 360µm o.d., 20µm i.d., 10µm tip i.d.) were used with application of 1500 V of liquid junction voltage and 250 °C of capillary temperature. For MS/MS analysis, data dependent acquisition (DDA) was employed with a top-12 mode at a resolving power of 17500 (at 200 m/z). A target automatic gain control (AGC) value of 1e6, and a maximum fill time of 60ms were used.

Protein identification

For protein identification of LC-MS/MS files, a protein database search was performed using the MASCOT search engine with 1% FDR. Carbamidomethylcysteine was set as fixed modification. Methionine oxidation, deamination, and N-terminal pyroglutamate were set as variable modifications. Proteome Discoverer (version 1.4, Thermo Scientific) was used for database search and data analysis.
