# Supplementary material for: A new ALK isoform transported by extracellular vesicles confers drug resistance to melanoma cells
Source: Mol Cancer. 2018 Oct 5;17:145. doi: 10.1186/s12943-018-0886-x (PMC6172729; doi:10.1186/s12943-018-0886-x)
Supplement: Supplementary file 3 — Supplementary Figures S1–S7. (ZIP 3175 kb) [file 12943_2018_886_MOESM3_ESM.zip › Figure S1.pdf]

Figure S1

```
                      Exon 16
                     ------->
ALK_NM_004304.4     GTGGTGGAGGTGGCTGGAATGATAACACTTCCTTGCTCTGGGCCGGAAAATCTTTGCAGG      60
A375X1              -----AGATCCAGGTAAACTGA--------------------------CAGCTCTGATCG      29
                          **    * *  * ***                           * ** **   *

ALK_NM_004304.4     AGGGTGCCACCGGAGGACATTCCTGCCCCCAGGCCATGAAGAAGTGGGGGTGGGAGACAA      120
A375X1              AGTCTGTCCTCATCACCCATCAGCCCACCTGGGACGACTGTCAGCAGCTGTTGGGGACTC      89
                    **  ** *  *      ***     * **  ** *       **  *  ** ** ***

ALK_NM_004304.4     GAGGGGGTTTCGGAGGGGGTGGAGGGGGGTGCTCCTCAGGTGGAGGAGGCGGAGGATATA      180
A375X1              ----------------TGCTGACCGGAGAAGAAAAACAACGGGTGCTCTTAGAGGCTAGA      133
                                     * **   ** *  *     **   ** *      **** ** *
                        Exon 17
                       ------->
ALK_NM_004304.4     TAGGCGG-------CAATGCAGCCTCAAACAATGACCCCGAAATGGATGGGGAAGATGGG      233
A375X1              AAGGCGGTGCGGGGCGATGATGGGCGCCCCACTCAACTGCCCAATGAA---GTCGATGCC      190
                     ******       * ***  *       ** * * *     *  **    *  ****
                                                                    Exon 18
                                                                   ------->
ALK_NM_004304.4     GTTTCCTTCATCAGTCCACTGGGCATCCTGTACACCCCAGCTTTAAAAGTGATGGAAGGC      293
A375X1              GCT---TTTCCCCTCGAGCGCCCAGACTGGGATTACACCACCCAGGCAGTGATGGAAGGC      247
                    * *   **   *      *       *  * *   * *  *      *************

ALK_NM_004304.4     CACGGGGAAGTGAATATTAAGCATTATCTAAACTGCAGTCACTGTGAGGTAGACGAATGT      353
A375X1              CACGGGGAAGTGAATATTAAGCATTATCTAAACTGCAGTCACTGTGAGGTAGACGAATGT      307
                    ************************************************************

ALK_NM_004304.4     CACATGGACCCTGAAAGCCACAAGGTCATCTGCTTCTGTGACCACGGGACGGTGCTGGCT      413
A375X1              CACATGGACCCTGAAAGCCACAAGGTCATCTGCTTCTGTGACCACGGGACGGTGCTGGCT      367
                    ************************************************************

ALK_NM_004304.4     GAGGATGGCGTCTCCTGCATTGTGTCACCCACCCCGGAGCCACACCTGCCACTCTCGCTG      473
A375X1              GAGGATGGCGTCTCCTGCATTGTGTCACCCacCCCGGAGCCACACCTGCCACTCTCGCTG      427
                    ************************************************************

ALK_NM_004304.4     ATCCTCTCTGTGGTGACCTCTGCCCTCGTGGCCGCCCTGGTCCTGGCTTTCTCCGGCATC      533
A375X1              ATCCTCTCTGTGGTGACCTCTGCCCTCGTGGCCGCCCtggtCCTGGCTTTCTCCGGCATC      487
                    ************************************************************

ALK_NM_004304.4     ATGATTGTGTACCGCCGGAAGCACCAGGAGCTGCAAGCCATGCAGATGGAGCTGCAGAGC      593
A375X1              ATGATTGTGTACCGCCGGAAGC--------------------------------------      509
                    **********************

ALK_NM_004304.4     CCTGAGTACAAGCTGAGCAAGCTCCGCACCTCGACCATCATGACCGACTACAACCCCAAC      653
A375X1              ------------------------------------------------------------      509
```

Figure S1 Alignment after Sanger sequencing of the 5′-RACE-cDNA fragments confirming the starting of ALK from exon 18 (green arrow). The asterisks indicate a perfect match with the reference sequence present in the NCBI database.
