# Supplementary material for: A new ALK isoform transported by extracellular vesicles confers drug resistance to melanoma cells
Source: Mol Cancer. 2018 Oct 5;17:145. doi: 10.1186/s12943-018-0886-x (PMC6172729; doi:10.1186/s12943-018-0886-x)
Supplement: Supplementary file 3 — Supplementary Figures S1–S7. (ZIP 3175 kb) [file 12943_2018_886_MOESM3_ESM.zip › Figure S2.pdf]

Figure S2

```
         Exon 18
           ↓
ALK     MEGHGEVNIKHYLNCSHCEVDECHMDPESHKVICFCDHGTVLAEDGVSCIVSPTPEPHLP 60
X1      MEGHGEVNIKHYLNCSHCEVDECHMDPESHKVICFCDHGTVLAEDGVSCIVSPTPEPHLP 60
        ************************************************************

ALK     LSLILSVVTSALVAALVLAFSGIMIVYRRKHQELQAMQMELQSPEYKLSKLRTSTIMTDY 120
X1      LSLILSVVTSALVAALVLAFSGIMIVYRRKHQELQAMQMELQSPEYKLSKLRTSTIMTDY 120
        ************************************************************

ALK     NPNYCFAGKTSSISDLKEVPRKNITLIRGLGHGAFGEVYEGQVSGMPNDPSPLQVAVKTL 180
X1      NPNYCFAGKTSSISDLKEVPRKNITLIRGLGHGAFGEVYEGQVSGMPNDPSPLQVAVKTL 180
        ************************************************************

ALK     PEVCSEQDELDFLMEALIISKFNHQNIVRCIGVSLQSLPRFILLELMAGGDLKSFLRETR 240
X1      PEVCSEQDELDFLMEALIISKFNHQNIVRCIGVSLQSLPRFILLELMAGGDLKSFLRETR 240
        ************************************************************

ALK     PRPSQPSSLAMLDLLHVARDIACGCQYLEENHFIHRDIAARNCLLTCPGPGRVAKIGDFG 300
X1      PRPSQPSSLAMLDLLHVARDIACGCQYLEENHFIHRDIAARNCLLTCPGPGRVAKIGDFG 300
        ************************************************************

ALK     MARDIYRASYYRKGGCAMLPVKWMPPEAFMEGIFTSKTDTWSFGVLLWEIFSLGYMPYPS 360
X1      MARDIYRASYYRKGGCAMLPVKWMPPEAFMEGIFTSKTDTWSFGVLLWEIFSLGYMPYPS 360
        ************************************************************

ALK     KSNQEVLEFVTSGGRMDPPKNCPGPVYRIMTQCWQHQPEDRPNFAIILERIEYCTQDPDV 420
X1      KSNQEVLEFVTSGGRMDPPKNCPGPVYRIMTQCWQHQPEDRPNFAIILERIEYCTQDPDV 420
        ************************************************************

ALK     INTALPIEYGPLVEEEEKVPVRPKDPEGVPPLLVSQQAKREEERSPAAPPPLPTTSSGKA 480
X1      INTALPIEYGPLVEEEEKVPVRPKDPEGVPPLLVSQQAKREEERSPAAPPPLPTTSSGKA 480
        ************************************************************

ALK     AKKPTAAEISVRVPRGPAVEGGHVNMAFSQSNPPSELHKVHGSRNKPTSLWNPTYGSWFT 540
X1      AKKPTAAEVSVRVPRGPAVEGGHVNMAFSQSNPPSELHKVHGSRNKPTSLWNPTYGSWFT 540
        ********:***************************************************

ALK     EKPTKKNNPIAKKEPHDRGNLGLEGSCTVPPNVATGRLPGASLLLEPSSLTANMKEVPLF 600
X1      EKPTKKNNPIAKKEPHDRGNLGLEGSCTVPPNVATGRLPGASLLLEPSSLTANMKEVPLF 600
        ************************************************************

ALK      RLRHFPCGNVNYGYQQQGLPLEAATAPGAGHYEDTILKSKNSMNQPGP    648
X1       RLRHFPCGNVNYGYQQQGLPLEAATAPGAGHYEDTILKSKNSMNQPGP    648
         ************************************************
```

Figure S2 . ALK sequencing results. The protein sequence of ALK expressed in A375X1 cells (blue) was aligned to the NCBI Reference Sequence (NM_004304.4).
The amino acidic substitution at position 489 is reported as a SNP (https://www.ncbi.nlm.nih.gov/projects/SNP/snp_ref.cgi?geneId=238).
