# Supplementary material for: A new ALK isoform transported by extracellular vesicles confers drug resistance to melanoma cells
Source: Mol Cancer. 2018 Oct 5;17:145. doi: 10.1186/s12943-018-0886-x (PMC6172729; doi:10.1186/s12943-018-0886-x)
Supplement: Supplementary file 3 — Supplementary Figures S1–S7. (ZIP 3175 kb) [file 12943_2018_886_MOESM3_ESM.zip › Figure S3.pdf]

Figure S3

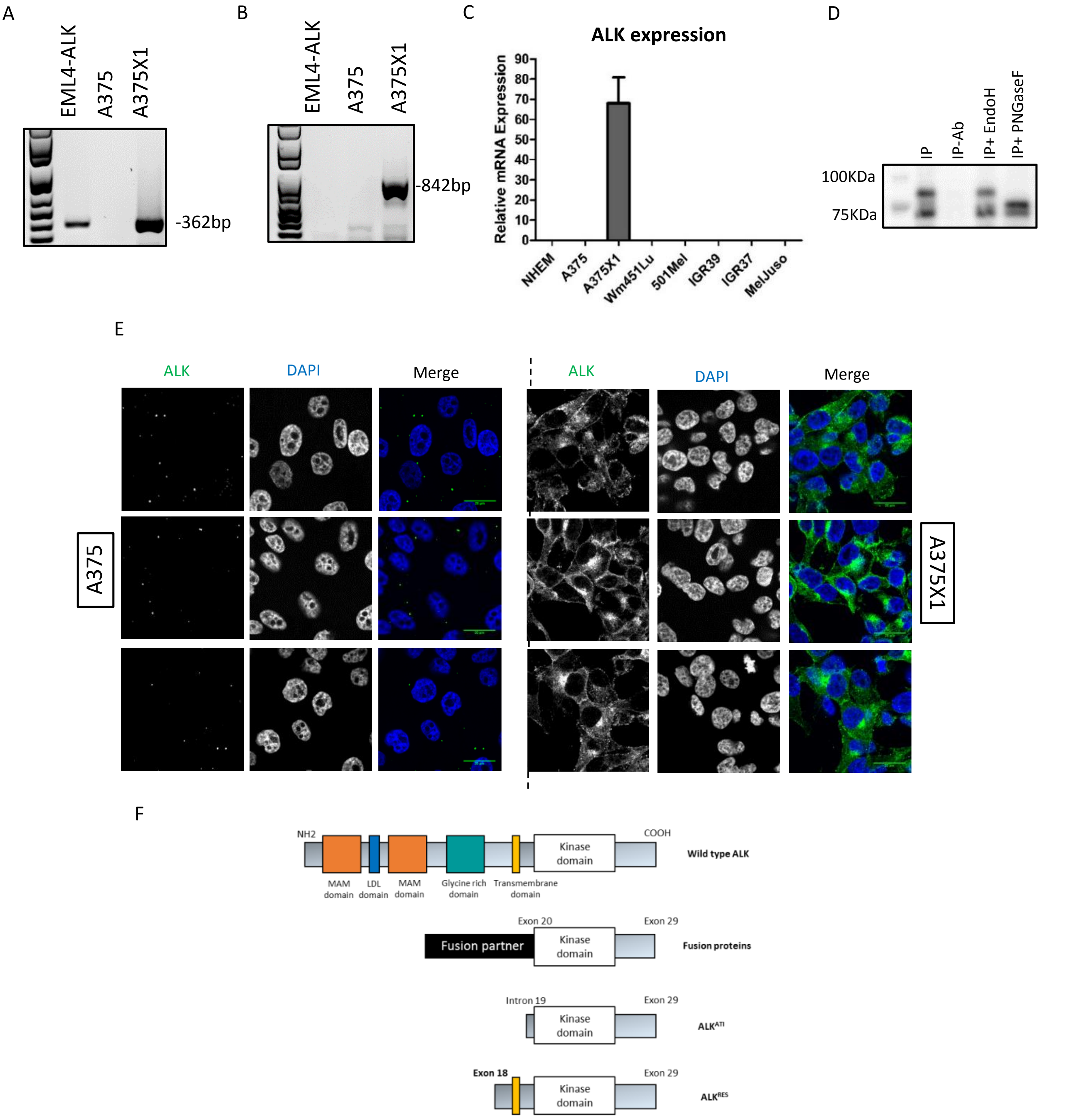

Figure S3. Characterisation of ALK^{RES} in drug-resistant A375X1 melanoma cells. **(A)** PCR amplification of ALK^{RES} in sensitive and resistant cells. EMLA4-ALK positive lung cancer cell line was used as positive control. **(B)** PCR amplification of the fusion MMLV-ALK is exclusively detected in the resistant cells. **(C)** Quantitative RT-PCR of ALK mRNA in different melanoma cell lines. Error bars represent the standard deviation of three technical replicates. **(D)** ALK^{RES} immunoprecipitation and subsequent western blot analysis yields two different protein bands. **(E)** Sensitive A375 and resistant A375X1 were fixed and stained for ALK. Images were captured by fluorescence confocal microscopy. Representative images of two biological replicates. Scale bar, 20µm. Blue: nucleus; green: ALK. **(F)** Schematic representation of different ALK proteins. The full length ALK contains two MAM domains, one LDL domain, a glycine-rich domain, a transmembrane domain and the intracellular kinase domain. In fusion proteins, the translocation often occurs at a common breakpoint in exon 20 of the ALK locus involving the entire kinase domain. The truncated ALK^{ATI} contains the exons 20–29 preceded by ~400 base pairs of intron 19. The ALK^{RES} protein identified in resistant melanoma cells contains 70 extracellular amino acids, the transmembrane domain and the whole cytoplasmic domain.
