# Supplementary material for: A new ALK isoform transported by extracellular vesicles confers drug resistance to melanoma cells
Source: Mol Cancer. 2018 Oct 5;17:145. doi: 10.1186/s12943-018-0886-x (PMC6172729; doi:10.1186/s12943-018-0886-x)
Supplement: Supplementary file 3 — Supplementary Figures S1–S7. (ZIP 3175 kb) [file 12943_2018_886_MOESM3_ESM.zip › Figure S4.pdf]

Figure S4

A

```
MGAIGLLWLL PLLLSTAAVG SGMGTGQRAG SPAAGPPLQP REPLSYSRLQ RKSLAVDFVV
PSXXXXXXXD LLLPPSSSEL KAGRPEARGS LALDCAPLLR LLGPAPGVSW TAGSPAPAEA
RTLSRVLKGG SVRKLRRAKQ LVLELGEEAI LEGCVGPPGE AAVGLLQFNL SELFSWWIRQ
GEGRLRIRLM PEKKASEVGR EGRLSAAIRA SQPRLLFQIF GTGHSSLESP TNMPSPSPDY
FTWNLTWIMK DSFPFLSHRS RYGLECSFDF PCELEYSPPL HDLRNQSWSW RRIPSEEASQ
MDLLDGPGAE RSKEMPRGSF LLLNTSADSK HTILSPWMRS SSEHCTLAVS VHRHLQPSGR
YIAQLLPHNE AAREILLMPT PGKHGWTVLQ GRIGRPDNPF RVALEYISSG NRSLSAVDFF
ALKNCSEGTS PGSKMALQSS FTCWNGTVLQ LGQACDFHQD CAQGEDESQM CRKLPVGFYC
NFEDGFCGWT QGTLSPHTPQ WQVRTLKDAR FQDHQDHALL LSTTDVPASE SATVTSATFP
APIKSSPCEL RMSWLIRGVL RGNVSLVLVE NKTGKEQGRM VWHVAAYEGL SLWQWMVLPL
LDVSDRFWLQ MVAWWGQGSR AIVAFDNISI SLDCYLTISG EDKILQNTAP KSRNLFERNP
NKELKPGENS PRQTPIFDPT VHWLFTTCGA SGPHGPTQAQ CNNAYQNSNL SVEVGSEGPL
KGIQIWKVPA TDTYSISGYG AAGGKGGKNT MMRSHGVSVL GIFNLEKDDM LYILVGQQGE
DACPSTNQLI QKVCIGENNV IEEEIRVNRS VHEWAGGGGG GGGATYVFKM KDGVPVPLII
AAGGGGRAYG AKTDTFHPER LENNSSVLGL NGNSGAAGGG GGWNDNTSLL WAGKSLQEGA
TGGHSCPQAM KKWGWETRGG FGGGGGGCSS GGGGGGYIGG NAASNNDPEM DGEDGVSFIS
PLGILYTPAL KVMEGHGEVN IKHYLNCSHC EVDECHMDPE SHKVICFCDH GTVLAEDGVS
CIVSPTPEPH LPLSLILSVV TSALVAALVL AFSGIMIVYR RKHQELQAMQ MELQSPEYKL
SKLRTSTIMT DYNPNYCFAG KTSSISDLKE VPRKNITLIR GLGHGAFGEV YEGQVSGMPN
DPSPLQVAVK TLPEVCSEQD ELDFLMEALI ISKFNHQNIV RCIGVSLQSL PRFILLELMA
GGDLKSFLRE TRPRPSQPSS LAMLDLLHVA RDIACGCQYL EENHFIHRDI AARNCLLTCP
GPGRVAKIGD FGMARDIYRA SYYRKGGCAM LPVKWMPPEA FMEGIFTSKT DTWSFGVLLW
EIFSLGYMPY PSKSNQEVLE FVTSGGRMDP PKNCPGPVYR IMTQCWQHQP EDRPNFAILL
ERIEYCTQDP DVINTALPIE YGPLVEEEEK VPVRPKDPEG VPPLLVSQQA KREEERSPAA
PPPLPTTSSG KAAKKPTAAE ISVRVPRGPA VEGGHVNMAF SQSNPPSELH KVHGSRNKPT
SLWNPTYGSW FTEKPTKKNN PIAKKEPHDR GNLGLEGSCT VPPNVATGRL PGASLLLEPS
SLTANMKEVP LFRLRHFPCG NVNYGYQQQG LPLEAATAPG AGHYEDTILK SKNSMNQPGP
```

B

```
MGAIGLLWLL PLLLSTAAVG SGMGTGQRAG SPAAGPPLQP REPLSYSRLQ RKSLAVDFVV
PSXXXXXXXD LLLPPSSSEL KAGRPEARGS LALDCAPLLR LLGPAPGVSW TAGSPAPAEA
RTLSRVLKGG SVRKLRRAKQ LVLELGEEAI LEGCVGPPGE AAVGLLQFNL SELFSWWIRQ
GEGRLRIRLM PEKKASEVGR EGRLSAAIRA SQPRLLFQIF GTGHSSLESP TNMPSPSPDY
FTWNLTWIMK DSFPFLSHRS RYGLECSFDF PCELEYSPPL HDLRNQSWSW RRIPSEEASQ
MDLLDGPGAE RSKEMPRGSF LLLNTSADSK HTILSPWMRS SSEHCTLAVS VHRHLQPSGR
YIAQLLPHNE AAREILLMPT PGKHGWTVLQ GRIGRPDNPF RVALEYISSG NRSLSAVDFF
ALKNCSEGTS PGSKMALQSS FTCWNGTVLQ LGQACDFHQD CAQGEDESQM CRKLPVGFYC
NFEDGFCGWT QGTLSPHTPQ WQVRTLKDAR FQDHQDHALL LSTTDVPASE SATVTSATFP
APIKSSPCEL RMSWLIRGVL RGNVSLVLVE NKTGKEQGRM VWHVAAYEGL SLWQWMVLPL
LDVSDRFWLQ MVAWWGQGSR AIVAFDNISI SLDCYLTISG EDKILQNTAP KSRNLFERNP
NKELKPGENS PRQTPIFDPT VHWLFTTCGA SGPHGPTQAQ CNNAYQNSNL SVEVGSEGPL
KGIQIWKVPA TDTYSISGYG AAGGKGGKNT MMRSHGVSVL GIFNLEKDDM LYILVGQQGE
DACPSTNQLI QKVCIGENNV IEEEIRVNRS VHEWAGGGGG GGGATYVFKM KDGVPVPLII
AAGGGGRAYG AKTDTFHPER LENNSSVLGL NGNSGAAGGG GGWNDNTSLL WAGKSLQEGA
TGGHSCPQAM KKWGWETRGG FGGGGGGCSS GGGGGGYIGG NAASNNDPEM DGEDGVSFIS
PLGILYTPAL KVMEGHGEVN IKHYLNCSHC EVDECHMDPE SHKVICFCDH GTVLAEDGVS
CIVSPTPEPH LPLSLILSVV TSALVAALVL AFSGIMIVYR RKHQELQAMQ MELQSPEYKL
SKLRTSTIMT DYNPNYCFAG KTSSISDLKE VPRKNITLIR GLGHGAFGEV YEGQVSGMPN
DPSPLQVAVK TLPEVCSEQD ELDFLMEALI ISKFNHQNIV RCIGVSLQSL PRFILLELMA
GGDLKSFLRE TRPRPSQPSS LAMLDLLHVA RDIACGCQYL EENHFIHRDI AARNCLLTCP
GPGRVAKIGD FGMARDIYRA SYYRKGGCAM LPVKWMPPEA FMEGIFTSKT DTWSFGVLLW
EIFSLGYMPY PSKSNQEVLE FVTSGGRMDP PKNCPGPVYR IMTQCWQHQP EDRPNFAILL
ERIEYCTQDP DVINTALPIE YGPLVEEEEK VPVRPKDPEG VPPLLVSQQA KREEERSPAA
PPPLPTTSSG KAAKKPTAAE ISVRVPRGPA VEGGHVNMAF SQSNPPSELH KVHGSRNKPT
SLWNPTYGSW FTEKPTKKNN PIAKKEPHDR GNLGLEGSCT VPPNVATGRL PGASLLLEPS
SLTANMKEVP LFRLRHFPCG NVNYGYQQQG LPLEAATAPG AGHYEDTILK SKNSMNQPGP
```

Fig. S4. ALK consensus sequence in which the highlighted peptides in yellow are the ones detected by MS after in-gel digestion. **(A)** Peptides identified in the upper band of the ALK IP (higher molecular weight); **(B)** Peptides identified in the lower band of the IP. Blue highlighting indicates alternating exons; Red highlighting indicates amino acids encoded across a splice junction.
