# Supplementary material for: A new ALK isoform transported by extracellular vesicles confers drug resistance to melanoma cells
Source: Mol Cancer. 2018 Oct 5;17:145. doi: 10.1186/s12943-018-0886-x (PMC6172729; doi:10.1186/s12943-018-0886-x)
Supplement: Supplementary file 3 — Supplementary Figures S1–S7. (ZIP 3175 kb) [file 12943_2018_886_MOESM3_ESM.zip › Figure S5.pdf]

Figure S5

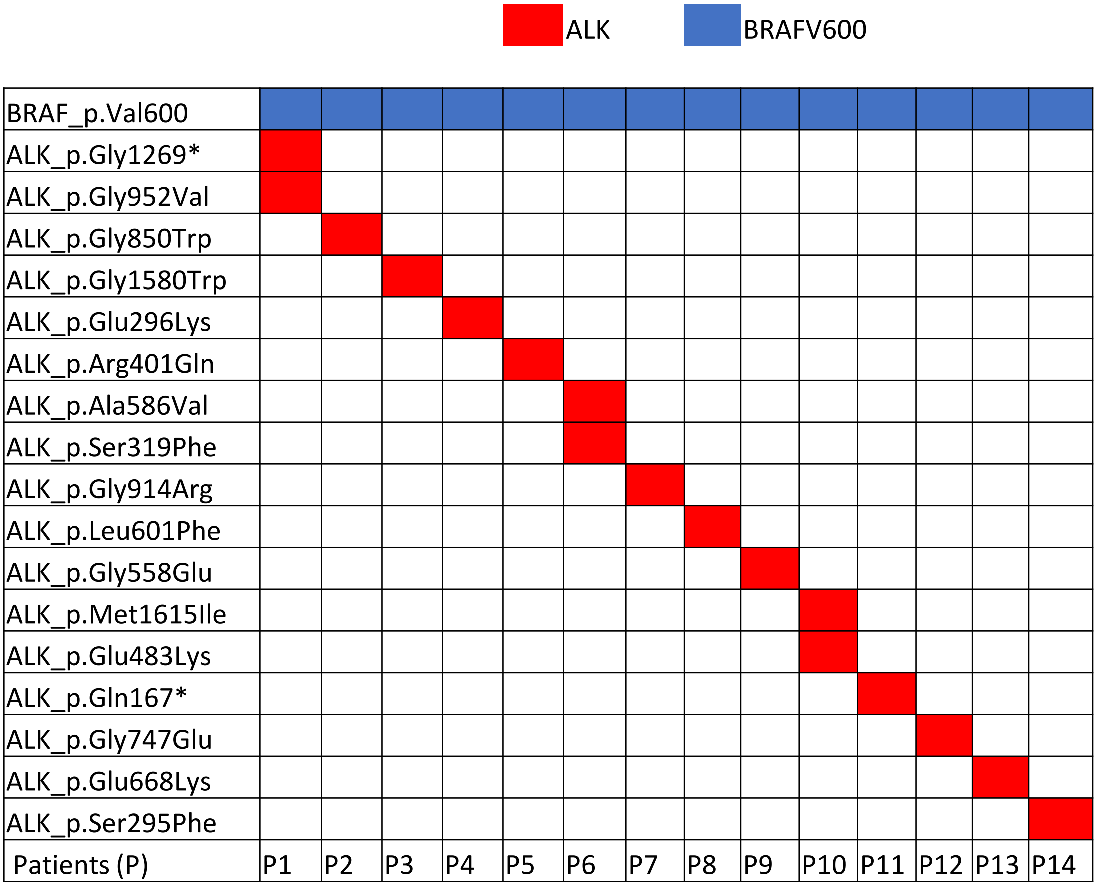

Figure S5. Missense mutations present in ALK together with $BRAF^{V600}$ in melanoma patients listed in the TCGA database. The mutations have been extracted with a Python (v. 2.7.9) script from MuTect2 TCGA vcf-files.
