# Supplementary material for: A new ALK isoform transported by extracellular vesicles confers drug resistance to melanoma cells
Source: Mol Cancer. 2018 Oct 5;17:145. doi: 10.1186/s12943-018-0886-x (PMC6172729; doi:10.1186/s12943-018-0886-x)
Supplement: Supplementary file 3 — Supplementary Figures S1–S7. (ZIP 3175 kb) [file 12943_2018_886_MOESM3_ESM.zip › Figure S6.pdf]

Figure S6

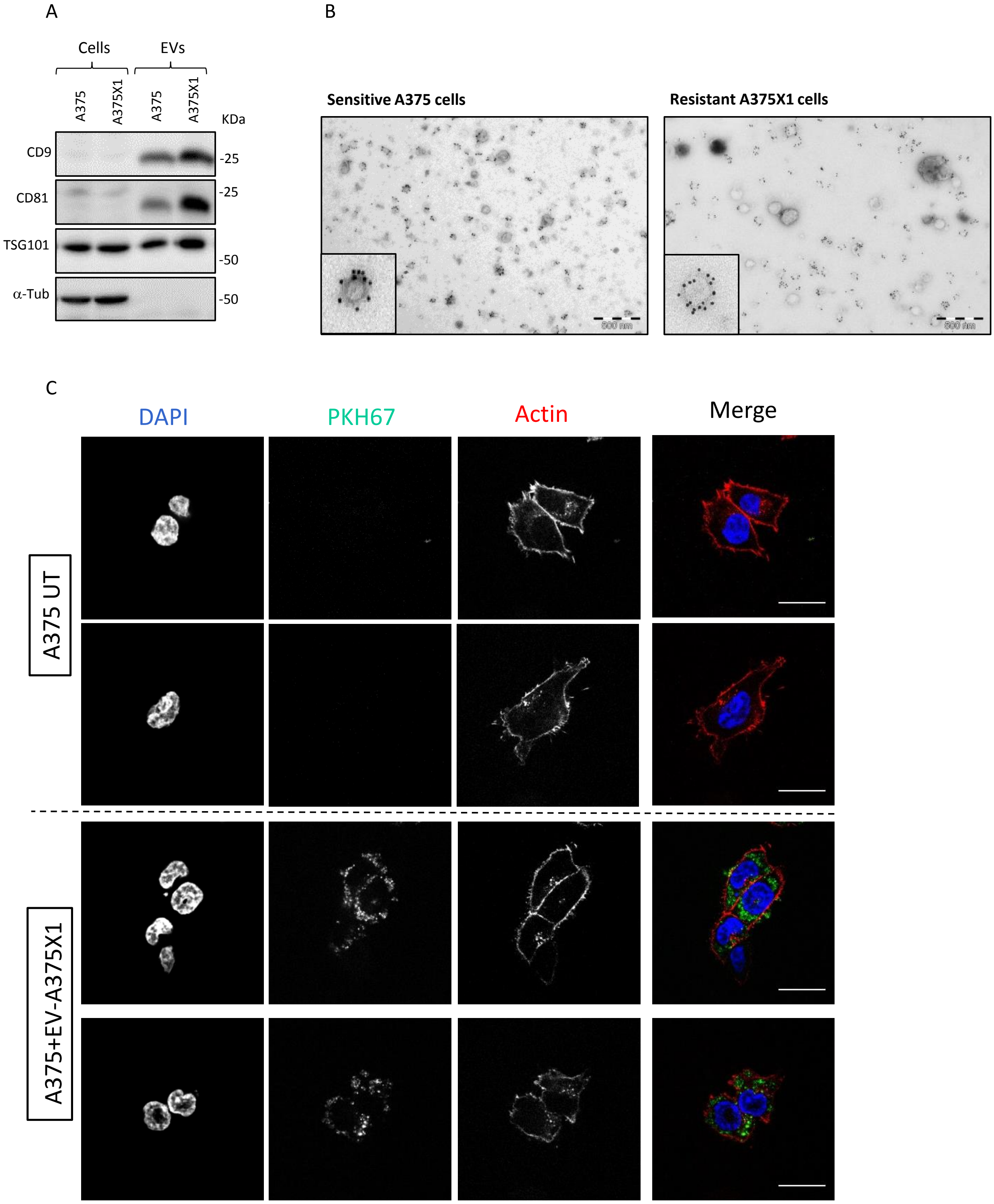

Figure S6. Characterisation of extracellular vesicles isolated from both sensitive and resistant melanoma cells. **(A)** Western blot analysis of 20μg lysates from sensitive A375 and resistant A375X1 cells and corresponding EVs. Results are shown for one representative of two biological replicates. **(B)** Transmission electron microscopy pictures of CD63 immunogold labelled EVs isolated from both cell lines. Results are shown for one representative of two biological replicates. **(C)** Resistant EVs were labeled with PKH67 before OptiPrep cushion separation. A375 cells were co-cultured with 10μg of labeled EVs and fixed after 24 hours. Upper panel, untreated sensitive A375 cells; Lower panel, sensitive A375 co-cultured with “resistant EVs”. Images are shown for one representative of two biological replicates. Blue: nucleus; red: actin; green: EVs.
