# Supplementary material for: A new ALK isoform transported by extracellular vesicles confers drug resistance to melanoma cells
Source: Mol Cancer. 2018 Oct 5;17:145. doi: 10.1186/s12943-018-0886-x (PMC6172729; doi:10.1186/s12943-018-0886-x)
Supplement: Supplementary file 3 — Supplementary Figures S1–S7. (ZIP 3175 kb) [file 12943_2018_886_MOESM3_ESM.zip › Figure S7.pdf]

Figure S7

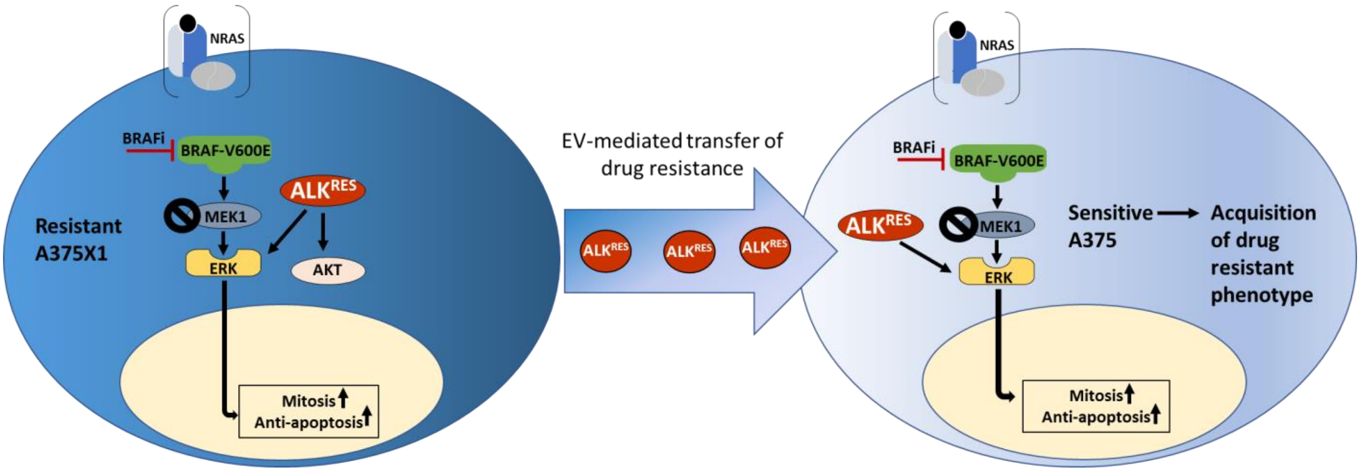

Figure S7. Model summarising the ALK^{RES} mediated transfer of drug resistance: overexpressed ALK^{RES} is secreted into extracellular vesicles which, in turn, can be taken up by sensitive cells. Once in sensitive cells, ALK is functional and can activate the MAPK pathway mediating drug resistance transfer.
